# Generation of In Vivo‐Inspired 3D Collagen Models for Guided Tumor Invasion In Vitro

**DOI:** 10.1002/cpz1.70280

**Published:** 2026-01-13

**Authors:** Stijn den Daas, Gert‐Jan Bakker, Diede van Ens, Eleni‐Andria Grosu, Manon Vullings, Lianne Beunk, Peter Friedl, Katarina Wolf

**Affiliations:** ^1^ Department of Medical BioSciences, Radboud Institute for Molecular Life Sciences Radboud University Medical Center Nijmegen The Netherlands

**Keywords:** collagen hydrogel, cell migration, guidance models, multiphoton microscopy, track generation by laser ablation

## Abstract

Porous collagen hydrogels are widely used to model dynamic cell‐extracellular matrix (ECM) interactions relevant to inflammation, wound healing, and cancer invasion. To improve the physiological relevance of such assays, it is essential to incorporate architectural ECM characteristics identified in vivo that may affect the mechanical and molecular mechanisms of cell migration, including ECM geometry, alignment, and dimensionality. We present detailed, step‐by‐step protocols for generating three collagen‐hydrogel‐based migration assays that integrate structural guidance cues, either as cleft‐like deformable gaps or tunnel‐like tracks, including track generation by multiphoton (MP) laser ablation. For this application, practical guidance on laser setup and integration in commonly used MP microscopes is provided. We include example applications to compare the migratory behavior of HT1080 fibrosarcoma cells, applied both as single‐cell suspensions and as pre‐formed spheroids, in these spatially controlled models. The data indicate that migration efficiency increases with the presence of guidance cues, highlighting the importance of such cues for modeling invasive cell behavior in 3D. These protocols provide a standardized yet adaptable framework for researchers studying ECM‐guided cell migration and evaluating therapeutic strategies that target cell motility. © 2026 The Author(s). Current Protocols published by Wiley Periodicals LLC.

**Basic Protocol 1**: Under‐collagen assay

**Basic Protocol 2**: 3D interface assay

**Basic Protocol 3**: 3D tissue track assay

**Support Protocol 1**: Imaging of guidance models by confocal microscopy

**Support Protocol 2**: Excitation beam adjustment

**Support Protocol 3**: Generation of stacks of long tracks by three common microscope types

## Introduction

**Figure 1:**
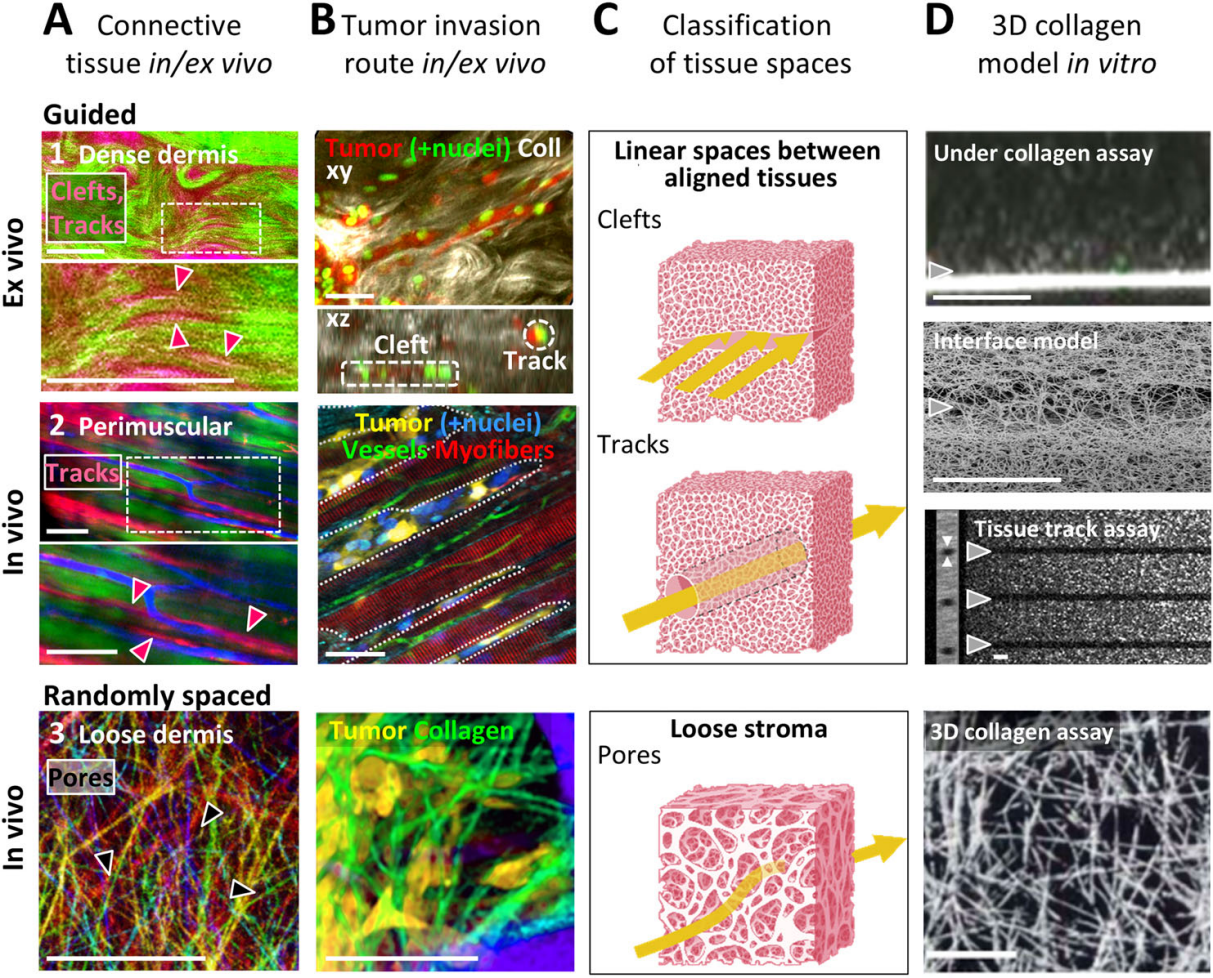
Guided versus porous tissue geometries in vivo and the design of tumor‐guiding collagen models in vitro. (**A**) Tumor‐free interstitial connective tissues with distinct geometries in vivo or ex vivo. **1**, Human dermis showing collagen‐ (green) and dextran‐filled clefts (red; arrowheads). **2**, Murine perimuscular tissue with linear interstitial tracks (dextran in red) between myofibers (green) and vessels (blue); arrowheads indicate tracks. In **1** and **2**, dotted rectangles mark zoomed‐in areas in lower row. **3**, Collagen‐rich upper dermis (mouse scalp), with randomly organized, porous spaces (black; arrowheads); depth layers are color‐coded. (**B**) Tumor invasion along the corresponding native tissue geometries shown in **A**. Colors denote tissue structures and invading tumors as indicated. (**C**) Classification of the respective geometries, with yellow arrows indicating cell migration trajectories. (**D**) Corresponding in vitro collagen models mimicking the geometries in **A‐C**; arrowheads mark preferred tumor cell migration routes. (**A and B**) Multiphoton microscopy; (**D**) confocal reflection microscopy except for image depicting the interface model (electron microscopy). Scale bars, 50 µm (**A and B**); 10 µm (**D**). Reprinted images, in part adapted, are with permission from John Wiley & Sons, https://www.wiley.com (Friedl et al., 2007; **A**, bottom); Taylor & Francis Ltd, http://www.tandfonline.com (Weigelin et al., 2012; **B**, middle)**;** Springer Nature, https://link.springer.com (Beunk et al., 2022; **D**, second from bottom); and the American Association for Cancer Research, https://aacrjournals.org (Friedl et al., 1997; **D**, bottom). The images in **C** were created in BioRender (den Daas, S., 2025; https://BioRender.com/z54p909).

Migration of cells through and along tissues rich in extracellular matrix (ECM) is a fundamental biological process intrinsic to nearly all cells in multicellular organisms. Cell migration is first observed during embryogenesis and continues throughout life, contributing to wound healing, immune surveillance (for selected cell types), and, when dysregulated, cancer invasion and metastasis. Each migration process is initiated and maintained by molecular and physical guidance cues from the extracellular environment, which control cell polarization, cytoskeletal activity, and interaction with tissue structures (Paksa et al., 2016; Te Boekhorst et al., 2016). Physical extracellular guidance cues are primarily provided by (i) three‐dimensional, collagen‐rich fibrillar ECM networks that can be loosely organized and porous or aligned and dense, and (ii) linear, aligned tracks situated between anatomical structures such as blood vessels, collagen bundles, nerves, or muscle fibers (Fig. 1A; Paul et al., 2017; Ray et al., 2022; Weigelin et al., 2012). Some of these linear tracks may also correspond to previously described fluid‐flow conduits in human tissues (Benias et al., 2018). Our recent studies, conducted both *in vivo* using live‐anesthetized mice and *ex vivo* in human dermal tissue, have demonstrated that aligned tracks comprise a variety of guiding interfaces—such as inner 2D surfaces, tunnels, or clefts—that are preferentially exploited by rapidly moving immune cells, but also by individually and/or collectively invading tumor cells (Fig. 1B; Weigelin et al., 2012; Weigelin et al., 2021). Understanding the mechanical and molecular principles underlying interface‐guided cell migration in the context of cancer and immunotherapy research requires the use of *in vitro* interface models that accurately recapitulate *in vivo* conditions of tissue organization and cell migration behaviors. Such interface models reduce the reliance on costly, technically demanding, and time‐intensive *in vivo* experiments.

To replicate these guiding environments *in vitro*, as observed *in vivo* through intravital microscopy, researchers have developed a variety of three‐dimensional (3D) models, such as synthetic microfluidic channels or discontinuous scaffolds of various geometries (Hulshof et al., 2017; Schwarz et al., 2016) and ECM‐based models such as collagen hydrogels, but also incorporating basement membrane or alginate extracts. These latter models are designed to include anisotropic, aligned architectures. Examples include aligned 3D collagen networks generated through fibroblast‐mediated mechanical strain (Ray et al., 2017) or ice‐templating techniques (Campbell et al., 2017); double‐layer gel assays (Uroz et al., 2024); and collagen microtrack platforms fabricated using polydimethylsiloxane (PDMS) stamping (Mosier et al., 2023). In our laboratory, we have developed and utilized several collagen‐hydrogel‐based systems that, in addition to randomly distributed pores, incorporate structured guidance features of varying architectures. These include cleft‐forming 3D hydrogel lids, cleft‐like interfaces, and collagen‐ablated linear tracks (Fig. 1C and 1D; Beunk et al., 2022; Beunk et al., 2023; Ilina et al., 2020). Here, we provide detailed, step‐by‐step protocols for generating these models. Several of these methods involve complex procedures that could not be fully described in the Methods section of previous publications.

**Figure 2:**
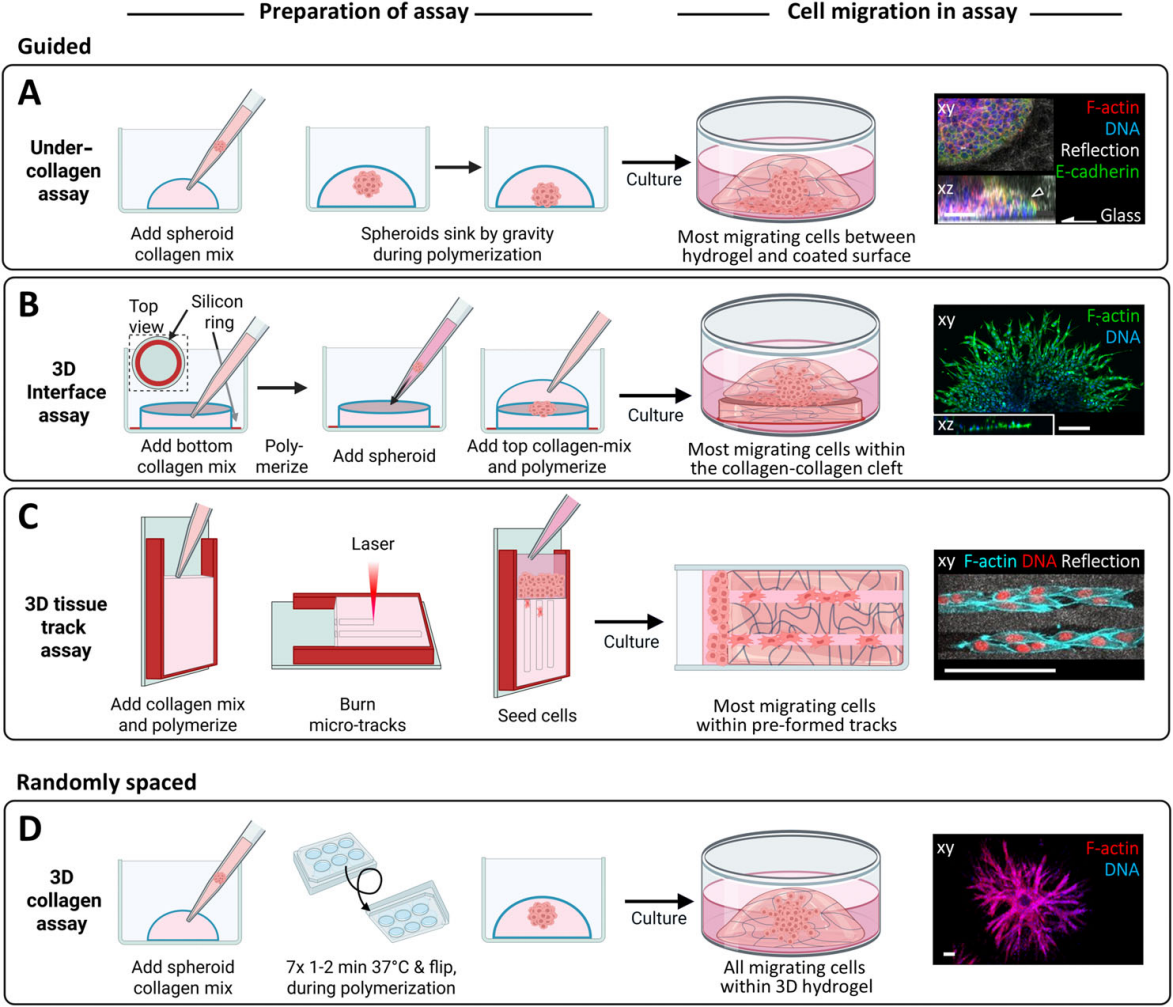
Overview of protocol steps for the generation of guided (**A‐C**) and unguided (**D**) collagen‐based migration models and outcomes. (**A‐D**) The cartoons at left outline the key preparation steps for the assays depicted, and those at right show the respective resulting migration patterns if steps are executed correctly. Gray curved lines in the cartoons at right depict collagen fibrils. The images at far right each show a respective microscopic image depiction of each migration model. All image bars, 100 µm. All cartoons were created in BioRender (den Daas, S., 2025; https://BioRender.com/beamh2y). Reprinted microscopic images, in part adapted, are with permission from Springer Nature, https://www.nature.com (Ilina et al., 2020; **A**), and Elsevier, https://www.elsevier.com (Lehmann et al., 2017; **D**).

Here, we describe protocols for the generation of three 3D collagen‐based guidance models with distinct geometries and types of applications (Figs. 1D and 2). Basic Protocol 1 outlines two alternative approaches for establishing an *under‐collagen assay*, in which a cleft is formed between a rigid cell culture substrate and a softer 3D collagen matrix. This cleft serves as a guidance interface for migrating cells. Basic Protocol 2 focuses on creating a *collagen‐collagen interface* or “sandwich” model by horizontally layering two dense 3D collagen hydrogels. This configuration allows cellular migration into distinct compartments of the model: the interfacial cleft and the adjacent randomly organized collagen matrix. Basic Protocol 3 presents a *linear 3D confining tissue track assay*—which enables cell migration within linear collagen‐free tunnels using advanced multiphoton laser ablation. We provide a detailed explanation of the underlying principles of track generation, as well as three accompanying support protocols. Support Protocol 1 provides guidance for optimizing confocal imaging of cells within the collagen matrices; Support Protocol 2 details the calibration and tuning of the excitation beam for precise track ablation; and Support Protocol 3 outlines brand‐specific solutions for optimal image acquisition and track stacking using various microscopy systems. These collagen‐based guidance models offer broad experimental utility, enabling the analysis of how tissue guidance cues influence cell sorting, cell‐matrix interactions, migration efficiency, and responses to migration‐inhibiting compounds (see Understanding Results). The information provided here should empower researchers to replicate and apply these guidance models with high reliability, robustness, and reproducibility.

### Collagen Base Substrate for 3D Cell‐Collagen Lattices

As a general note, the base substrate used in all guidance models described in Basic Protocols 1‐3 is a porous, three‐dimensional (3D) type I collagen hydrogel. This hydrogel can be prepared according to the recipe provided in the Reagents and Solution section at varying final concentrations, for example 1.7, 4, 5, or 6 mg collagen/ml (Table 1), depending on the required matrix density. Briefly, in preparing the collagen matrix, all stock solutions should be kept on ice, and low‐retention pipet tips are recommended to minimize volume loss. The collagen mixture—referred to here as the *collagen mix—*is formed by carefully but thoroughly combining minimal essential medium (MEM), NaOH, viscous acidic type I collagen monomers (isolated from rat tail or bovine skin), and cell culture medium (see Reagents and Solutions section and Table 1). For more information on the preparation of collagen mixes and 3D cell‐collagen cultures, please refer to Svoren et al. (2023). The collagen mix is then dispensed as droplets into a culture well plate and polymerized at 37°C in a humidified, 5%‐10% CO_2_ incubator. Cells for embedding in collagen can be prepared either as single‐cell suspensions in culture medium (Maaser et al., 1999; Svoren et al., 2023) or as pre‐formed spheroids in medium generated via the hanging‐drop method or using low‐adhesion culture plates (Veelken et al., 2017; Ware et al., 2016; Wolf et al., 2013). The cell or spheroid suspensions can then be added to the collagen mix by replacing the indicated volume of the cell culture medium. For details of spheroid generation required in Basic Protocols 1 and 2, see Veelken et al. (2017). Once polymerized, collagen‐cell constructs should be fully overlaid with culture medium. Empty wells in the plate should be filled with 1× PBS to maintain a humidified environment and prevent dehydration. Importantly, all model systems described in this protocol can be analyzed either via live‐cell time‐lapse microscopy, to monitor dynamic migration behavior, or by fixation, staining, and subsequent imaging as detailed in other studies from our laboratory (Beunk et al., 2022; Beunk et al., 2023; Ilina et al., 2020; Svoren et al., 2023; Veelken et al., 2017). These systems are compatible with various microscopy techniques, including brightfield, confocal, and multiphoton microscopy. Specific confocal imaging requirements for each guidance model are detailed in Support Protocol 1.

**Table 1:** Calculation Schemes for Collagen Mixes for Cell‐Collagen Cultures of Various Indicated Concentrations Used in These Protocols

	Collagen concentration			
Ingredients	1.7 mg/ml	4 mg/ml	5 mg/ml	6 mg/ml
10× MEM	50 µl	80 µl	80 µl	80 µl
1 N NaOH	4.5 µl	11 µl	13 µl	16 µl
Milli‐Q water	289.5 µl	342 µl	248 µl	154 µl
Rat tail collagen I	156 µl	367 µl	459 µl	551 µl
Culture medium (± cells)	500 µl	200 µl	200 µl	200 µl

The calculations are based on a rat tail collagen stock concentration of 10.9 mg/ml and an 1 ml final volume. For every new lot of collagen, the amount of NaOH required to achieve a pH of 7.3‐7.5 needs be tested. If live imaging experiments are performed on a microscope without a CO_2_ incubation system, HEPES may be added to the water as detailed in the recipe for collagen mix in Reagents and Solutions, below.

## Basic Protocol 1 UNDER‐COLLAGEN ASSAY

Under‐collagen assays are designed to mimic collapsed tissue clefts or inner surfaces bordered by linear anatomical structures such as collagen bundles, fascia, blood vessels, or myofibers. A key feature of this model is the formation of a flat, linear cleft between the rigid cell culture surface and an overlying 3D extracellular matrix (ECM) lattice. The density of the ECM can be adjusted to modulate the degree of physical confinement imposed on migrating cells (Ilina et al., 2020). Two principal approaches can be used to establish this assay: (1) the sinking‐down method (steps 1a‐3a), in which a pre‐formed spheroid is placed into a collagen solution and allowed to settle naturally onto the cell culture surface by gravity (Ilina et al., 2020); and (2) the precoating method (steps 1b‐7b), in which a glass or plastic culture surface is first coated with a thin ECM layer, the spheroid is positioned on this coated surface, and then a top ECM layer is added. Collagen, used for both precoating and the top layer, can in principle be substituted with other ECM components, such as basement membrane extract (Gritsenko et al., 2020). When properly executed, these approaches result in the formation of a well‐defined cleft beneath the ECM “lid,” along which cells predominantly migrate in a radial pattern outward from the spheroid.

### Materials

10× Minimum Essential Medium Eagle (MEM; Sigma, cat. no. M0275)
1 N NaOH (VWR, cat. no. NC2024371)
Viscous acidic type I collagen monomers (isolated from rat tail or bovine skin): Collagen I, high concentration (8‐11 mg/ml), rat tail (Corning, cat. no. 354249)
Milli‐Q‐purified water
Culture medium (see recipe)
Phosphate‐buffered saline (PBS; see recipe)

Multiwell cell culture plate suitable for microscopic imaging (e.g., Ibidi, cat. no. 81226)
Humidified 37°C, 5%‐10% CO_2_ incubator (ThermoFisher, series 8000 WJ, cat. no. 3429)

#### Sinking‐down method

1a Freshly prepare a spheroid‐containing collagen mix, possibly of varying collagen concentration, by carefully but thoroughly combining minimal essential medium (MEM), NaOH, viscous acidic type I collagen monomers (isolated from rat tail or bovine skin), and cell culture medium in the appropriate quantities, depending on the concentration of collagen desired (see article introduction, Reagents and Solutions recipe for collagen mix, and Table 1). Pipet 100 µl per well, containing one spheroid per aliquot, into a multi‐well (i.e., 12‐well) cell culture plate (Fig. 2A, left). Carefully position each spheroid in the center of the collagen droplet to ensure symmetrical embedding within the gel.
  Ensure that the collagen mix pipetted into each well contains only one spheroid. Embedding multiple spheroids in a single collagen gel may result in the generation of traction force between spheroids, leading to local alignment and stiffening of collagen fibrils. This effect can introduce additional, and potentially unwanted, guidance cues within the model, thereby compromising experimental control.
2a Place the plate in the humidified 37°C, 10% CO_2_ incubator, in the upright position, and incubate for ∼30 min.
  After sinking and reaching the rigid surface, cells migrate in a confined 2D manner along the cleft (Fig. 2A, right). This differs from the classical 3D assay (Fig. 2D), in which plates must be flipped during collagen polymerization to position the spheroid centrally within the 3D matrix, allowing true 3D migration. The key difference between the sinking‐down and 3D assay lies in the spheroid positioning by gravity alone.
3a Add culture medium to fully cover each collagen gel (e.g., ∼2 ml per well in a 12‐well plate), and incubate for 1 day to several days, depending on the cell type and desired readout.

#### Precoating method

1b Add acid monomeric collagen solution (20 µg/ml) to each plate well to fully coat the bottom (e.g., 150 µl per well in a 96‐well plate).
2b Incubate plate for 3 hr.
3b Aspirate the collagen solution and wash once with 1× PBS; the resulting pH shift induces the formation of small fibrils.
4b Gently resuspend the spheroid suspension, collect 1‐5 µl containing a single visible spheroid, and place it in the center of the precoated well.
5b Incubate for 2.5 hr to allow spheroid attachment.
6b Prepare fresh collagen mix, add to the well (e.g., 70 µl), and polymerize in the incubator for ∼30 min.
7b Overlay each gel with medium (e.g., ∼130 µl), and incubate as needed.

## Basic Protocol 2 3D INTERFACE ASSAY

Similar to under‐collagen assays, interface (or “sandwich”) assays model linear clefts between aligned tissue structures. Unlike in under‐collagen setups, here both cleft walls are made of collagen, offering architecture and stiffness closer to physiological conditions. We describe how to generate this assay by overlaying a polymerized collagen hydrogel with a second gel. As a related method, we also outline a 3D collagen surface assay, in which cells migrate on top of a single thick gel, that is useful as a control (Beunk et al., 2023).

### Additional Materials (also see Basic Protocol 1)

Silicone rings/squares: silicone isolators of 8‐9 mm diameter and 0.5 mm depth (GRACE Bio‐Labs, cat. no. GBL664501)

1 Place sterile silicone rings into a multiwell plate (e.g., 24‐ or 6‐well), one or more per well. Gently tap each ring with a pipet tip to seal it against the plate bottom; check seal quality by inspecting from underneath.
2 Prepare the collagen mix for the bottom collagen gel (as in Basic Protocol 1, step 6b) and pipet 50 µl into each silicone ring (Fig. 2B).
  Prepare only enough collagen mix for the bottom gel to avoid premature polymerization of the top layer.
3 Polymerize the collagen droplet by incubating the plate for ∼30 min in a humidified 37°C, 5%‐10% CO_2_ incubator and then remove from the incubator.
4 Pipet one spheroid (1‐5 µl medium) gently onto the center of the polymerized bottom hydrogel, without piercing the collagen.
  Because of the small volume, removing excess medium is usually unnecessary. However, if spheroids stick inside the pipet tip and only medium is released, removing excess medium by pipetting it off or using sterile filter paper in the periphery of the small droplet may help.
5 For the interface assay, prepare cell‐free collagen mix for the top gel and pipet it gently over the spheroid and bottom collagen gel.
  The spheroid may shift slightly but should remain on the bottom collagen gel.
6 Place the well plate with interface assay samples (and controls without the top gel, if used) in the incubator for ∼30 min.
7 Add medium to fully cover the collagen gels (e.g., ∼2 ml per well in a 12‐well plate) and incubate the cell‐collagen cultures.
  Occasionally, the top collagen layer may detach from the bottom, typically due to expired collagen, insufficient polymerization time, low collagen concentration (i.e., <2 mg/ml), or a wet surface of the bottom gel. To minimize the likelihood of the latter, keep the bottom collagen layer outside the incubator for 20‐30 min (between steps 3 and 5) to allow partial evaporation, which improves adhesion between the two layers. However, note that surface drying can alter collagen structure, increasing fibril density and stiffness. For reproducibility, decide at the outset of the experimental series whether or not to apply this drying step consistently.

## Basic Protocol 3 3D TISSUE TRACK ASSAY

The tissue track model in vitro mimics linear, tube‐like paths of varying geometries found between collagen bundles, myofibers, or vessel‐lining fibers in vivo (Beunk et al., 2022; Ilina et al., 2011; Weigelin et al., 2012). Generation of laser‐ablated microtracks within collagen is more complex than the previous two assays and requires a three‐step, 48‐hr preparation (Fig. 2C): first, prepare a silicone‐based 3D glass chamber filled with collagen; second, perform laser ablation of the collagen; and third, add cells on top and allow them to migrate into the tracks. Proper execution allows monitoring and quantification of guided migration within linear collagen‐bordered tunnels.

### Materials

Silicone (Heraform type A+B, white and orange components; Kulzer, cat. no. 64600982)
70% (v/v) ethanol (EtOH)
1:1 (v/v) mix of paraffin (Sigma, cat. no. 76244‐1KG)/petroleum jelly (Vaseline; Sigma, cat. no. 16415‐1KG), melted at 70°C before use
Cell suspension: e.g., 0.5 × 10^6^ mesenchymal or epithelial tumor cells/ml
Isopropanol
Collagen mix (Table 1), ideally as 6 mg/ml collagen

**Figure 3:**
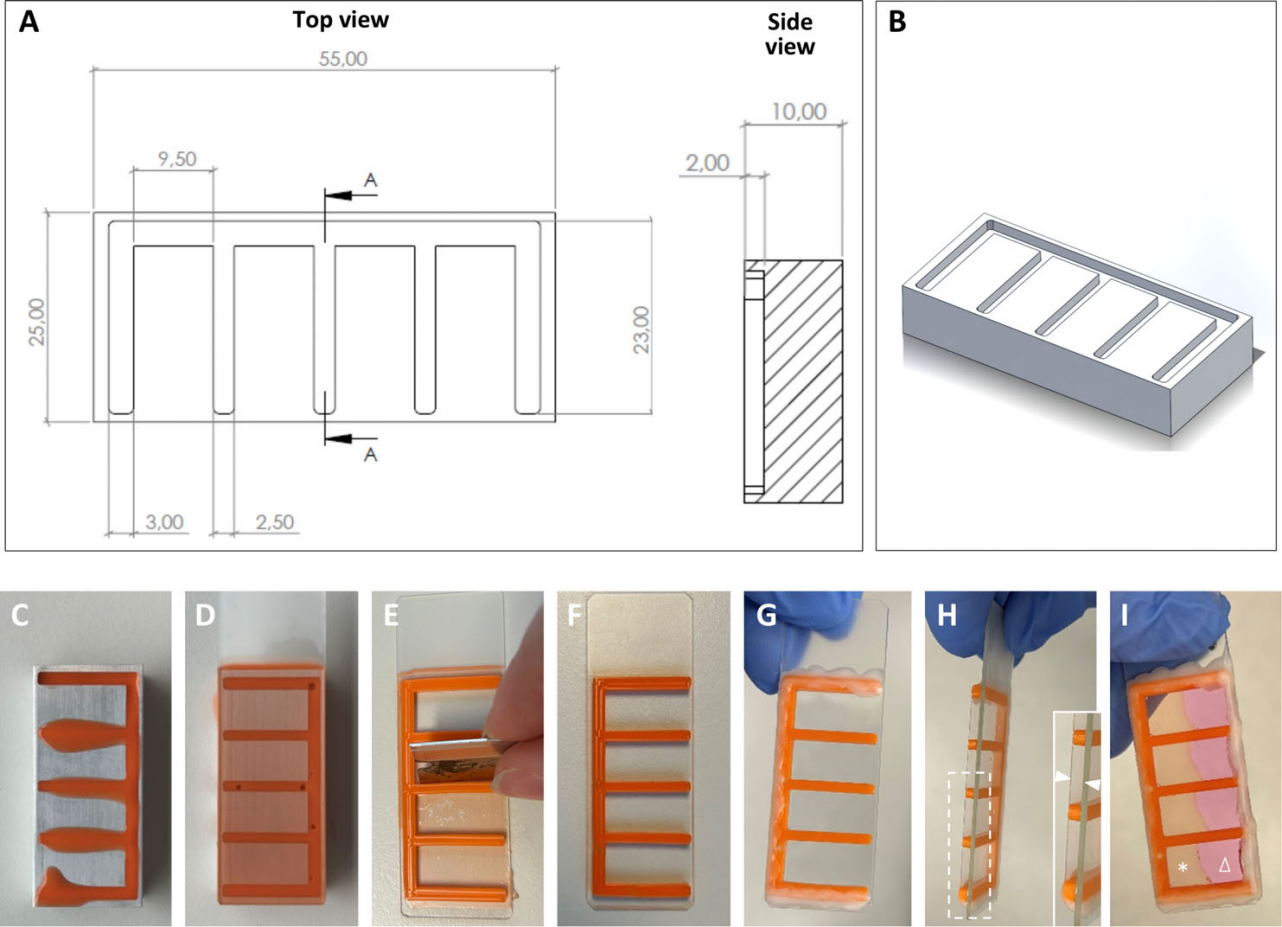
Fabrication process for the silicone chamber used in the 3D tissue track assay. (**A**) Technical drawing of the metal mold, depicting the lengths, widths, and depths in millimeters in front and side view. (**B**) 3D representation of the drawing of the metal stamp. The respective .STEP (Supporting Information File 1) and .STL files (Supporting Information File 2) for the generation of the stamp as used in this work are provided as Supporting Information, to enable fabrication of such stamps via additive (e.g., 3D printing) or subtractive (e.g., milling) manufacturing. (**C‐I**) Images depicting the process of generating the silicone chambers, supporting the descriptions in Basic Protocol 3, steps 1‐9. In **H**, the dotted rectangle depicts the location of the zoom‐in inset at right, showing the 2‐mm depth of the generated chamber indicated by the two arrowheads. In **I**, the asterisk indicates the polymerized collagen matrix; and the triangle denotes the overlaid medium that, after track generation, will be exchanged with a cell suspension.

Metal molds to form U‐shaped 3D chambers of 2 mm thickness and ∼0.4 ml volume (Fig. 3A and 3B; prepared in house; we obtained these from the Technical Support Group, Radboud University, FSW, made to specification)
3‐ml syringe (BD Plastipak, REF309658)
Glass microscope slides (VWR, cat. no. 631‐1553)
Scalpel (Dahlhausen, cat. no. 1100000510)
Coverslips: 24 × 50‐mm (no. 1.5) coverglasses (VWR, cat. no. 631‐0147)
Humidified 37°C, 10% CO_2_ incubator
Multiphoton microscope (TriMScope II, Miltenyi Biotec, Germany, or similar, as described in Support Protocol 3)
Computer and appropriate image acquisition software

#### Preparation of a 3D silicone chamber filled with collagen

The collagen‐filled chamber should ideally be prepared the day before the track‐generation procedure described in steps 10‐15 is performed.

1 Combine the white and orange silicone components in a 1:1 ratio using a syringe (e.g., 1 ml each for a single mold chamber) and mix thoroughly.
2 Using a syringe, transfer ∼1 ml of the mixture into each mold (Fig. 3C).
3 Cover each filled mold with a glass microscope slide and press firmly to adhere the silicone to the slide (Fig. 3D).
4 Allow the silicone mix to cure and fully solidify at room temperature for 20‐30 min.
5 Carefully lift one side of the glass slide to detach the silicone from the mold, and then remove any excess silicone using a scalpel or blade (Fig. 3E; extra silicone can be seen in some cells of the mold in Fig. 3F).
6 Clean both coverslip and silicone‐containing glass slide with 70% ethanol, dry them, and allow the ethanol to fully evaporate (Fig. 3F).
7 Place the coverslip on top of the chamber. Using a brush, seal the bottom and sides with 1:1 paraffin/Vaseline mixture, melted at 70°C, leaving the top of the multi‐cell silicone chamber open (Fig. 3G and 3H).
8 Prepare collagen mix (see recipe), and pipet 200 µl into each cell of the multi‐cell chamber to fill them around halfway. Place chamber in the humidified 37°C, 5%‐10% CO_2_ incubator and allow collagen mix to polymerize for ∼15 min.
9 Fill the chamber completely with medium, avoiding air bubbles. Seal with liquid paraffin/Vaseline (Fig. 3I) and store overnight at 4°C.

#### Laser‐generated collagen ablation

**Figure 4:**
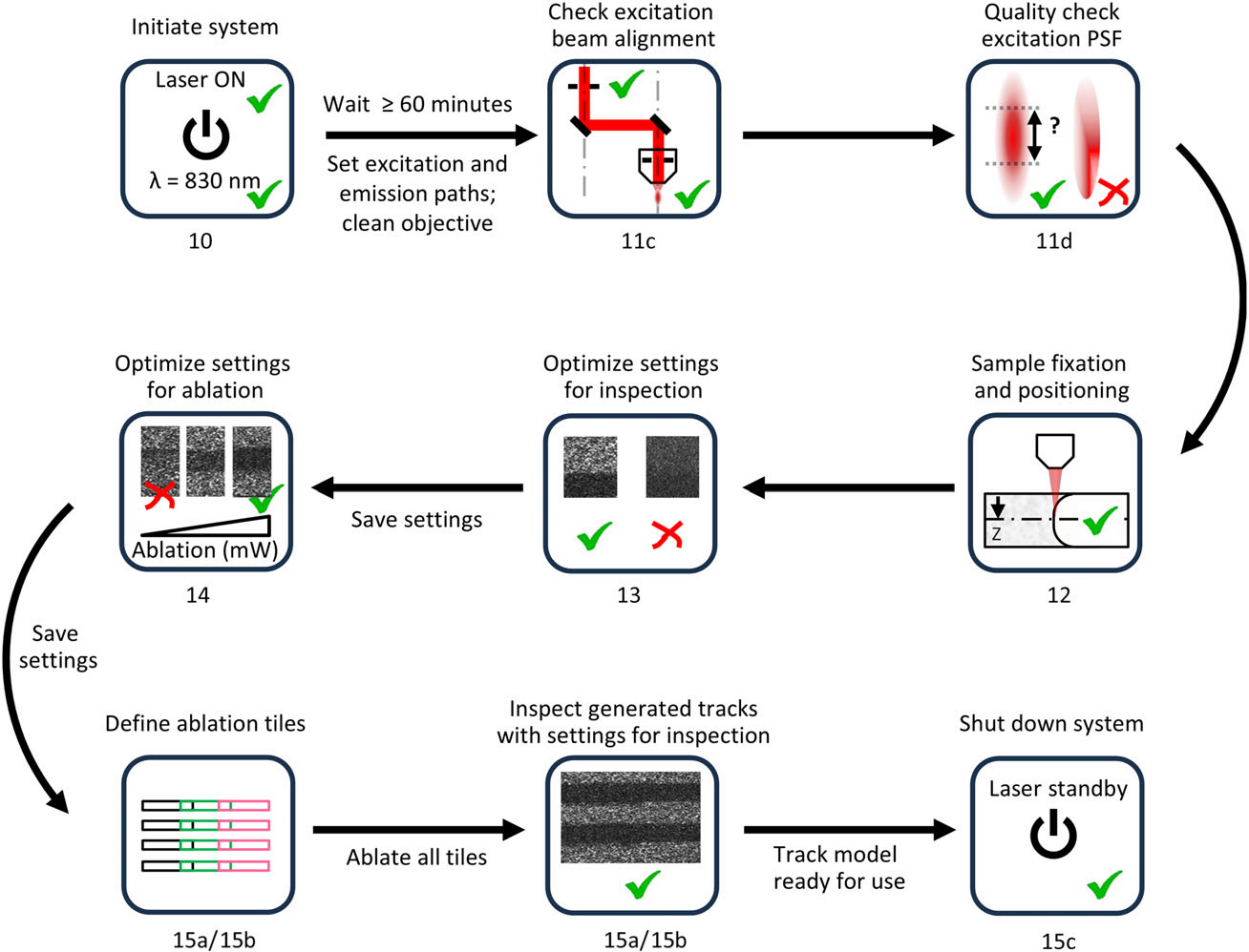
Overview of the workflow for laser‐generated collagen ablation to create the tissue tracks. The flow scheme serves as a visual complement to the detailed instructions provided in Basic Protocol 3, steps 10‐15. The numbers 10‐15 beneath each icon refer to the corresponding protocol steps.

**Figure 5:**
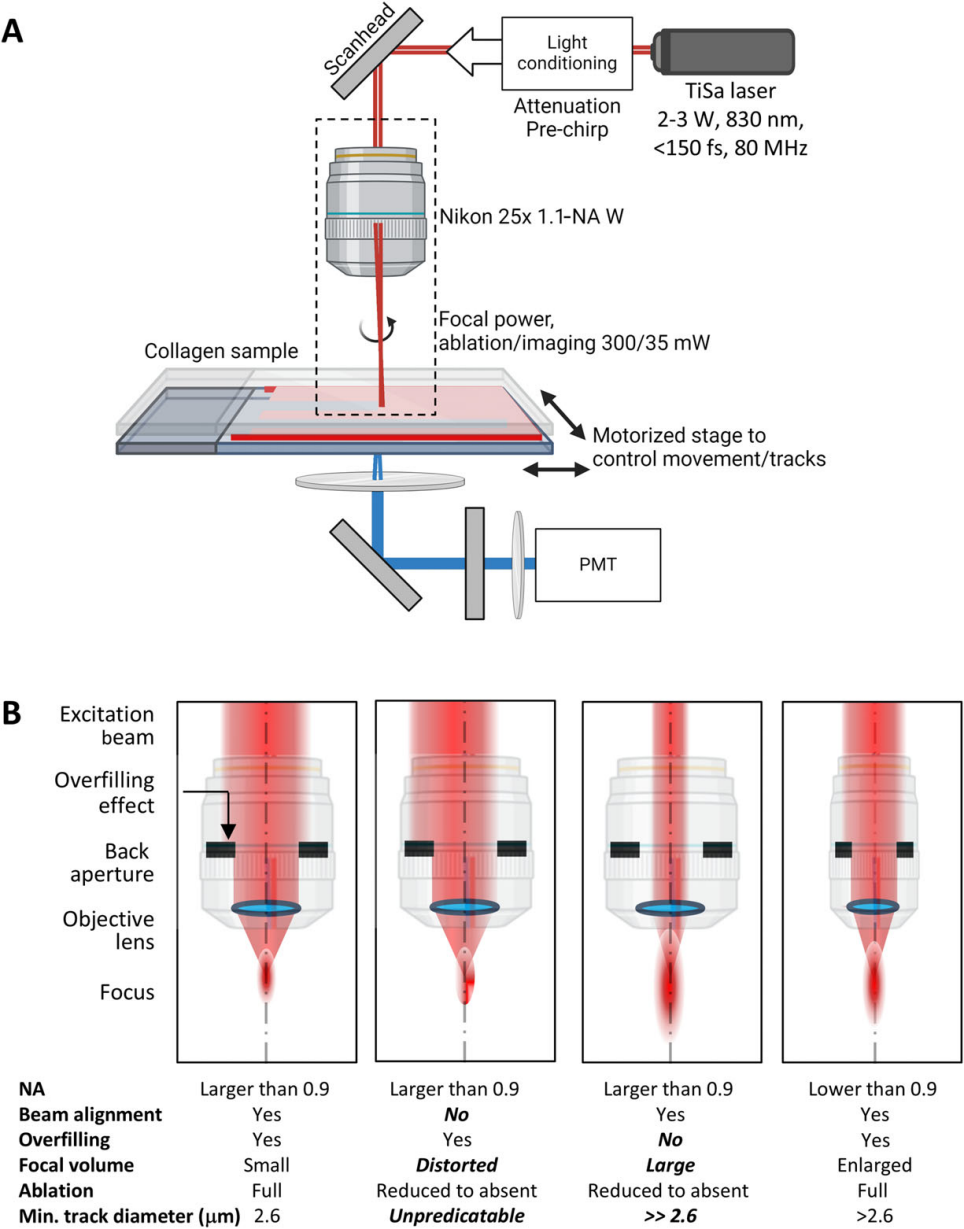
Principle of tissue track assay generation by multiphoton microscopy. (**A**) Multiphoton microscope setup for cutting and inspection of collagen‐free tracks in 3D collagen matrix by laser ablation. Excitation light is attenuated and pre‐chirped to compensate for microscope‐induced dispersion prior to entering the microscope scanhead. Laser beam input (red line) and output (blue line) for the visualization of collagen by SHG, respectively. The dotted rectangle shows the area of the zoomed regions in **B**, depicting an enlarged distance between objective and sample for clarity of the cartoon. SHG emission is collected in forward direction, filtered and detected using an PMT. (**B**) Principle of laser beam adjustment for focused track ablation. Overviews depicting excitation beam alignment (dotted line) and beam diameter enlargement over the diameter of the back aperture (“overfilling”) for the creation of small focal volumes. The image at left depicts the optimal settings where the beam travels through the center of the back aperture to concentrate the excitation power into the smallest possible focal spot to enable burning of defined tracks. All other cartoons show sub‐optimal settings. Terms in boldface italic in lower row indicate erroneous settings that will reduce or even avoid focused track ablation. Min., minimal. The cartoon in **A** was created in BioRender (den Daas, S., 2025; https://BioRender.com/opeya42).

Laser‐mediated track ablation in polymerized collagen using two‐photon (2P) microscopy (Figs. 4 and 5A) is an advanced approach reliant on several key factors. The six‐step procedure below (summarized in Fig. 4) details the microscope setup for focused multi‐track generation and inspection. The main outcomes are collagen‐depleted tracks bordered by a collagen interface with defined height, width, and length. Once continuous tracks with in‐vivo‐like dimensions are created, tube‐guided cell migration experiments and microscopic monitoring can be performed.

*CAUTION*: During track generation, 8‐40 times higher excitation energy arrives at the sample under the microscope objective than during imaging. Depending on your setup's laser safety features, extra precautions may be needed. Consult your local laser safety officer and follow all recommended safety measures before proceeding.

10 Initiate the microscope system:
  a. 60 min before aligning the excitation path, turn on the laser system. Then, start the microscope, computer, and acquisition software.
  b. Set up the microscope for track ablation and SHG imaging:
    i. Set laser wavelength to 830 nm and open the shutter.
    ii. Optimize excitation path for high 830‐nm transmission.
    iii. Adjust detection path for second‐harmonic generation (SHG) emission at 415 nm, preferably in the forward direction (see Commentary).
    iv. Use a high‐numerical‐objective (high‐NA), long‐working‐distance water objective (20‐25×, ≥0.95 NA).
  Proteins have very high single‐photon absorption in the UV range, and therefore, two‐photon‐absorption‐mediated ablation of collagen is most efficient at relatively short wavelengths within the near‐infrared range (Vogel & Venugopalan, 2003). In this protocol, and commonly in our lab, we use an 830‐nm wavelength for precise track ablation as well as to generate strong SHG signals with excellent signal‐to‐noise ratio (Hall et al., 2014; Beunk et al., 2022).
11 Validate the quality of the excitation point spread function (PSF) under the microscope objective:
  Track ablation requires precise focusing of the excitation beam into the sample, with the objective's PSF nearing its theoretical size and symmetrical shape along the optical axis. Therefore, proper alignment of the excitation beam position and diameter at the objective's back aperture is critical (Fig. 5B; Table 2). Although this step need not be done every time ablation is performed, we recommend validating the PSF on a regular basis. Additionally, all optical elements, especially the objective, must be in optimal condition.
  a. Clean the objective's pupil lens following the manufacturer's cleaning protocol.
  b. If available, adjust the objective's correction collar to match the sample coverglass thickness (e.g., 0.17 mm).
  c. Follow the manufacturer's instructions for excitation beam adjustment, including periodic beam alignment and optimization of beam position and diameter, after tuning to the excitation wavelength of 830 nm. Also see Support Protocol 2, parts 1 and 2.
  d. Assess the excitation focus quality by measuring and analyzing the PSF using fluorescent beads (Bakker et al., 2022; Theer et al., 2014). Check that the PSF full width at half maximum (FWHM) in lateral directions is within 125% and in axial direction is within 150% of the theoretical limits based on the objective's numerical aperture, the excitation wavelength, and the size of the fluorescent beads (as calculated in the analysis program created by Theer et al., 2014).
  e. Verify that the PSF is symmetrical along the optical axis, with minimum and maximum FWHM values in the imaging plane differing by <25%.
  f. Ensure the PSF shows no abnormal irregularities or extra maxima as the final quality check.

**Table 2:** Common Pitfalls, Possible Causes, and Solutions in Collagen Model Preparation and Tissue Track Generation and Visualization

Pitfall	Possible cause	Solution
Collagen hydrogel: no or incomplete polymerization	pH of collagen mix too acid or too alkalic; collagen reagent is past expiration date	Pay attention to expiry date; titrate pH within a range of 7.0‐7.5; provide sufficient time to polymerize.
Collagen hydrogel: air bubbles	Ingredients are mixed too fast	Mix gently, but efficiently.
Collagen hydrogel: heterogenous fibril formation	Insufficient or overly slow mixing of ingredients leading to local pre‐polymerization	Have all ingredients on ice and, when adding them successively, mix swiftly and thoroughly; use low‐retention pipet tips.
Under‐collagen and interface assay: spheroid “swims” to the side	The spheroid was added together with too much medium; mechanical friction occurred during handling	Reduce amount of medium around placed spheroid; handle gently.
Interface assay: top gel detaches from bottom gel	Bottom gel too hydrated, or incomplete collagen polymerization	See above. Dry the bottom gel outside the incubator for 20‐30 min.
Tissue track assay: successive tracks do not overlap perfectly (see Fig. 6C)	Instability of sample or sample fixation on the microscope stage leading to drift. Low stage positioning precision.	Fix sample on stage with clamps. Remove wax remnants from the sample bottom. Fix microscope stage; compensate for repetitive stage errors. Change the order of tiles during the ablation process.
Tissue track assay: less defined or incomplete tracks are generated	Excitation path misalignment; too low ablation power; laser pulse length too long. Objective or coverglass dirty.	Adjust excitation path/beam diameter; reduce collagen density; compensate for pulse dispersion; adapt track burning power to objective type and laser pulse length; clean objective and coverglass.
Tissue track assay: SHG signal for track visualization is low	Backscatter signal is used; misalignment of excitation or emission path; low excitation power; short pixel dwell time.	Use a GaAsP detector; use forward detection with a high‐NA condenser; align paths, increase excitation power/pixel dwell time/line averaging.
Tissue track assay: laser mode locking failure during ablation	Back‐reflection of laser light into the laser head.	Slightly adjust the excitation path; install an optical (Faraday) isolator.

**Figure 6:**
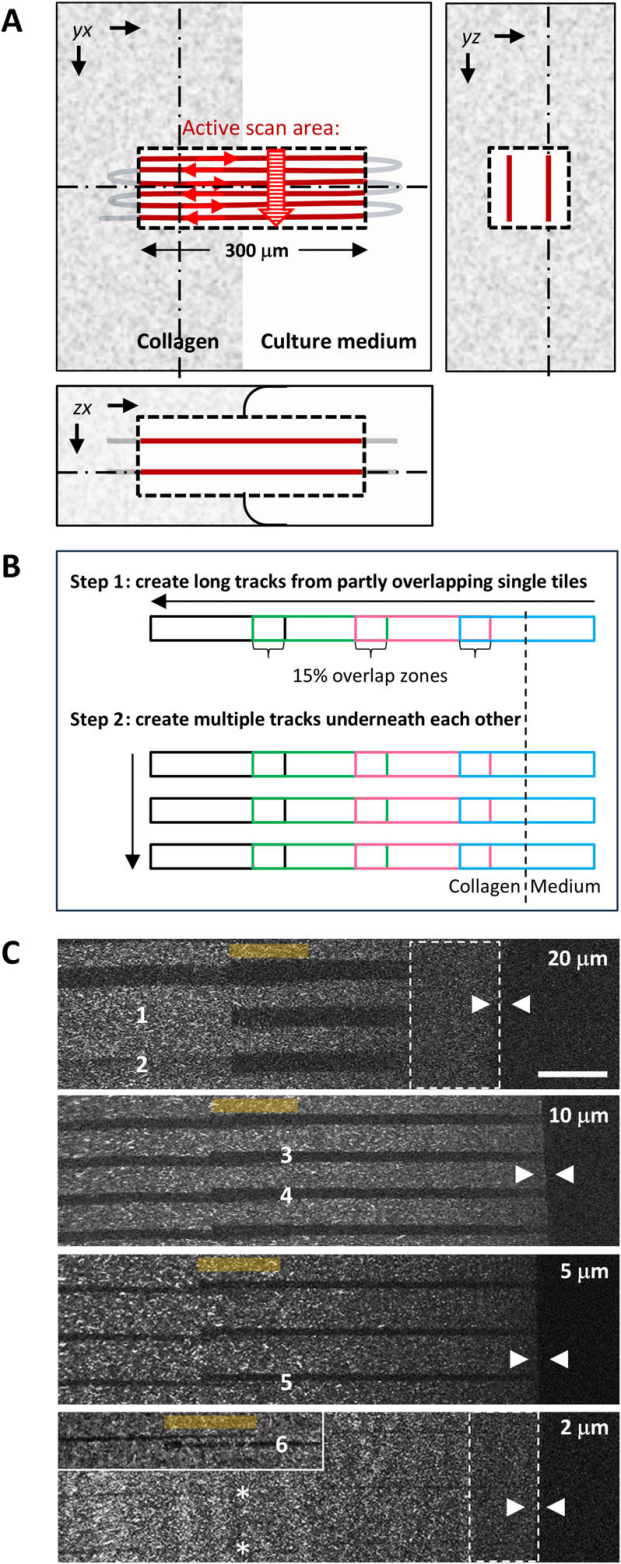
Performance and quality control during track ablation. (**A**) 3D depiction of laser scanning pattern for the generation of a 5‐µm track tile at the collagen‐medium interface in the silicone chamber. Note that ablation dimensions are not depicted at scale. *yx* panel: the scanning laser beam (brown) is activated inside the ablation tile region (dashed square). Horizontal red arrows indicate the direction of the scanning beam; striped red arrow indicates direction of tile generation. The side panels show orthogonal ablation planes positioned along the dotted lines in the *xy* panel. Brown lines, ablation planes. The lower panel shows the collagen meniscus between the two glass plates and the preferred positioning of the ablated track in the center of the collagen gel. (**B**) Stepwise procedure for generating long (step 1) and multiple (step 2) tracks. Arrows indicate the direction of track generation. (**C**) Depiction of multiple generated tracks at various indicated diameters in µm, together with pitfalls, such as sample drift in the axial (1, 2) or lateral direction causing partly (3, 4) or complete (5, 6) separation of adjacent ablated tiles. Also shown are faulty track end positions where interruption of forward migration is anticipated: by sample drift (5, 6) or by incomplete laser ablation (*). Dotted areas indicate zones of intact tracks for undisturbed entry of migrating cells (see Video 1) which, however, show weak contrast, presumably due to mild imaging artifacts. Orange lines indicate tile overlap zones, and arrowheads point to the medium‐collagen interface. Scale bar, 50 µm.

12 Correctly position the sample:
  a. Clean the microscope coverglass and sample slide bottom with lens paper and isopropanol, and then mount on the microscope stage (Fig. 5A).
  b. Bring the objective in working distance to the sample and add immersion water.
  c. Focus the collagen‐medium interface at the center of the objective.
13 Define the settings for collagen sample inspection using SHG:
  a. Set scanner parameters: pixel size ∼0.33 µm, image size ∼300 µm × 300 µm (adjust zoom or scan field), pixel dwell time 1–2 µs, and line averaging 1.
    The adjustment of the scanner settings is brand specific, and the suggested dimensions, pixel size, and pixel dwell time should be used as a guideline; see Support Protocol 3 for additional information.
  b. Begin with an excitation power of ∼25 mW under the objective. Refer to Support Protocol 2, part 3, for instructions on calibrating the power under the objective.
  c. Start live mode.
  d. Adjust the detector offset to ensure no zero‐value pixels appear in the background.
  e. Adjust the detector gain until the SHG signal from collagen fibrils is visible; stop increasing gain once image quality no longer improves.
  f. Position the sample so the collagen‐medium interface aligns parallel to the direction of tile generation (Fig. 6A, large vertical red arrow).
  g. Adjust imaging depth so the focal plane is at the lowest point of the collagen gel meniscus, about halfway into the sample chamber (Fig. 6A, lower panel), or somewhat closer to the coverslip, to ensure that the track plane can be reached during the life imaging experiment.
    At this position, the collagen‐medium interface runs parallel to the z axis (Fig. 6A, lower panel), ensuring the cleanest track entrance
  h. Optimize gain and excitation power to clearly contrast collagen and medium, keeping excitation power below ∼35 mW for high‐NA (>0.9) objectives.
    Aim for a signal‐to‐noise ratio ≥3, adding line averaging if needed.
  i. Save these “inspection” microscope settings for reuse after collagen ablation.

14 Prepare the microscope settings needed for track ablation:
  a. The goal is to ablate collagen locally, creating tracks of set cross‐sections (e.g., 2 × 2, 5 × 5, 10 × 10, or 20 × 20 µm^2^) and ∼300 µm in length; here, we detail the creation of 5 × 5 × 300 µm tracks. The goal of this step is to find the optimal laser ablation power; therefore, the laser power might be increased stepwise from 200 to ∼300‐400 mW until a satisfactory laser ablation result is obtained.
  b. Reduce the *xy* scan area to 5 µm × 300 µm (Fig. 6A, left), set line averaging to 1, and adjust pixel dwell time to ∼20 µs; this laser exposure ablates collagen 2.6 µm deep (Fig. 5B and Beunk et al., 2022).
  c. Set *z*‐stack mode with two planes at +2 µm and –2 µm from the focal point.
    These positions have been chosen empirically and depend on instrument type and settings. Take into account that the ablation area will be slightly larger than the selected scan area.
  d. Start by increasing excitation power to ∼200 mW for track ablation; run ablation, save settings as “ablation,” and then reload the “inspection” settings to check results.
    In live mode with “fine z‐adjustment,” a cleared area appears in xy and yz with its contrast close to that of the collagen‐medium interface (see Fig. 6C); similar track width and depth confirms satisfactory ablation settings.
  e. If ablation contrast is low or invisible, increase excitation power by 50 mW, move stage 30 µm vertically, and retry ablation.
  f. Track ablation settings for other track sizes must be determined experimentally.
    Ablation power stays constant across track sizes if pixel size and dwell time are unchanged. Note that axial ablation spread is greater than lateral spread because of the shape of the PSF; therefore, reduce z‐stack positions by 1 µm on each side to compensate.
15 Generate multiple long tracks by repeating laser ablation steps across different locations. For most applications, 300‐µm tracks are too short and one track is not sufficient; therefore, automate ablation for the generation of long and multiple tracks using the microscope's tiling or navigator mode.
  a. Generate long tracks by stitching together multiple ablation regions:
    i. Load the “ablation” settings in the microscope software.
    ii. Activate tiling mode and set tile overlap to 15%.
    iii. Define the number of ablation regions in a row to form one extended track (e.g., *x* = 4, *y* = 1 for four tracks with three overlapping regions along the track; see Fig. 6B).
      Most microscope software lets you adjust tile numbers in x and y directions by dragging region corners or entering integer values for x and y.
    iv. Position the tile region with the first tile halfway outside the collagen‐medium interface and the rest inside the collagen (Fig. 6A).
    v. Ablate all tiles.
    vi. Reload the “inspection” settings and verify that there is a continuous, well‐overlapping track.
  b. Next, extend the procedure to stack multiple long tracks vertically at regular intervals (Fig. 6B and 6C), following your microscope software's guidelines (Support Protocol 3).
  c. Shut down system.

#### Adding cells to monitor for migration

Add cells within 0‐24 hr after ablation. If cell seeding is planned for the following day, store the track‐containing chamber at 4°C and equilibrate in the incubator for 30‐60 min before adding cells.

16 Prepare a cell suspension according to the experimental design.
17 Remove the wax from the chamber top with a blade and aspirate the existing medium.
18 Add 200 µl of cell suspension onto the collagen (the chamber should be completely filled).
19 Incubate vertically for 60 min to let cells settle onto the collagen and to allow adjustment of the pH in the medium. Re‐seal with paraffin/Vaseline mix and return to horizontal position.
20 Incubate for the desired time, then fix, stain, and image; or proceed with live imaging from the onset of migration (see Video 1).

**Video 1:** Spontaneous migration of HT1080 fibrosarcoma cells into collagen‐based tissue tracks. Tissue tracks within a collagen lattice pretreated with the MMP inhibitor GM6001 were generated using preset laser settings of 2 µm (top) or 20 µm (bottom) width and ∼800 µm length. HT1080 cells labeled with the nuclear marker H2B‐eGFP (red) migrated into these tracks. Combined brightfield and fluorescence imaging was used to visualize microcracks, cell bodies, and nuclei at 4‐min intervals over ∼19 hr of observation. Video is reprinted and adapted with permission from Springer Nature, https://link.springer.com (Beunk et al., 2022).

## Support Protocol 1 IMAGING OF GUIDANCE MODELS BY CONFOCAL MICROSCOPY

Here, we provide practical tips and recommendations for imaging the three guidance models described in Basic Protocols 1‐3.

### Under‐collagen assay (Basic Protocol 1)

Imaging the under‐collagen assay is straightforward, as the cells predominantly migrate within the narrow cleft between the top collagen layer and the surface of the imaging well. This location places them well within the optical working distance of standard high‐magnification objectives (e.g., 40× or 60×) used in confocal microscopy.

- For monitoring migration, a time‐lapse acquisition using a tiled arrangement of *z*‐sectioned *xy* images typically suffices to track individual cells in the 2D plane.
- An orthogonal *xz* view can be reconstructed post acquisition to visualize the vertical position of the cells and the topography of the collagen interface, thereby illustrating the structural guidance cue (see Fig. 2A, right panels).

#### Important considerations

- The assay should remain in its original culture plate throughout imaging to prevent disruption of cell‐gel interactions.
- Fixed‐state imaging requires consideration of the slow diffusion of fixatives and antibodies through the dense collagen matrix.
- The imaging plate used must have an optically suitable thin bottom (e.g., no. 1.5 glass or plastic) to support high‐resolution confocal imaging.

#### 3D interface assay (Basic Protocol 2)

Cells typically migrate radially from the spheroid core into the cleft (the “interface”) formed between two collagen layers, while also invading the surrounding randomly organized 3D collagen matrix above and below (see Fig. 2B, right panels).

- For live‐cell imaging of cell movement specifically within the cleft, time‐resolved acquisition of single *xy* images or a limited number of *z*‐slices spanning the interface is usually sufficient. This enables efficient tracking of dynamic cell behavior within the guidance plane.
- To monitor cell positions throughout the full 3D matrix, including the interface and the adjacent collagen regions, tiled acquisition of *z*‐stacks from fixed samples is recommended.
- Acquired stacks can then be rendered in orthogonal views (*xz* or *yz*) to analyze both guided and unguided migration (Beunk et al., 2023).

##### Additional recommendations

- The cells in the interface assay are fully embedded, and therefore the gel can be detached from the culture dish for optimized staining and imaging.
- For improved visualization of deep‐lying cells, the gel can be cleared for reduced light scattering and improved imaging depth (Mai & Lu, 2024; Masselink et al., 2019), but also flipped during mounting, aligning the region of interest with the objective's working distance.
- Imaging quality can be further enhanced by using high‐NA objectives, appropriate imaging plates, and low‐scattering mounting media, which improve both resolution and signal quality.

#### 3D tissue track assay

In this model, cells migrate predominantly within the well‐defined, parallel‐oriented, collagen‐free tracks. A technical challenge of this assay is the relatively deep positioning of the ablation tracks, which may complicate imaging. For reliable visualization of fluorescent cell markers in either live or fixed cells, a 40× water‐immersion objective with a long working distance is recommended when using confocal microscopy (Beunk et al., 2022).

##### Additional recommendations

- For fixed samples, consider mild sample clearing to reduce scattering and improve *z*‐stack quality.
- Precheck laser penetration, fluorescence signal strength, and potential bleaching compensation at the desired depth before committing to long‐term imaging.
- Consider burning tracks at a closer (i.e., 300 µm) distance to the coverslip.

## Support Protocol 2 EXCITATION BEAM ADJUSTMENT

This protocol further outlines the steps explained in Basic Protocol 3, steps 11 and 13, providing details on the consecutive steps to calibrate and tune the excitation beam for precise track ablation, namely: (i) periodic beam alignment, (ii) adjustment of beam diameter and position after change of excitation wavelength, and (iii) control of correct laser power under the objective.

### Materials

Thermopile power meter (PM3, Coherent Corp., model 1098336)

#### Part 1: Inspection and correction of beam alignment

1 Use the software to park the excitation beam in the center of the scan area.
  If this function is not available, zoom in as much as possible to select the smallest region of interest in the center of the scan field. Set the scan frequency to ∼100 Hz and the image resolution to 512 × 512 pixels.
2 Remove the objective and replace it with a beam inspection target.
  A beam target can either be purchased from the manufacturer or improvised by drawing a crosshair target on a piece of paper. To use it, remove the objective and place the target in its position, ensuring that it is centered precisely over the opening in the empty objective slider or turret slot.
*CAUTION*: Laser safety note: The target must be securely fixed to the microscope and must fully cover the opening to prevent accidental laser exposure.
3 Set the laser power slider in the software to 0.
4 Open the excitation path shutters (if the beam is parked), or activate Live mode (if zoomed in) using the acquisition software.
5 Use an infrared viewer or a webcam without an infrared filter to inspect the beam on the target. For upright microscopes, a small mirror can assist with beam visualization. Gradually increase the laser power under the objective until the beam spot becomes visible.
  The spot generated by the continuously parked beam should remain in a stable position, with only minor on/off flickering due to image refresh.
6 A broad, unclipped beam with a Gaussian intensity profile should be visible on the target, with its peak intensity located near the center (±0.5 mm) of the target.
  If the beam is not centered, align the excitation path using the routine provided by the manufacturer. In some systems, this may be as simple as initiating an automated alignment procedure, whereas in others it requires manually adjusting the x and y screws on a beam mirror positioned >50 cm from the scanhead, while monitoring the beam on the target. If neither automated nor manual alignment achieves proper centering (deviation >0.5 mm), a service visit from the manufacturer should be considered. Additional overfilling of the objective back aperture can partly compensate for PSF degradation caused by beam misalignment. Before proceeding with track ablation (see part 2), follow the steps below to introduce additional overfilling (beyond the recommended 13.5% power loss from beam enlargement).
7 Keep the target and camera in place, and maintain the aligned excitation beam on the target for the procedures described in part 2 (steps 8‐14).

#### Part 2: Adjustment of the beam width at the objective back aperture

We describe here a procedure to optimize the beam diameter using power measurements under the objective as a readout. In most cases, an optimal two‐photon excitation PSF is obtained when the Gaussian laser beam overfills (i.e., is clipped by) the back aperture of the objective, reducing transmission through the objective by 13.5% (1/*e* ^2^). To determine this, we measure the power during beam diameter adjustment and compare it to the power measured with the smallest beam diameter (no overfilling). The optimal beam diameter corresponds to a relative transmission of 86.5% (100%–13.5%). In most commercial microscope systems, the beam diameter at the objective's back aperture can be adjusted via the acquisition software. The optimal setting depends on the microscope objective, excitation wavelength, and imaging depth. In custom setups, a telescope is often placed in front of the scan head; beam diameter at the back aperture can be modified by adjusting the telescope's exit beam divergence or diameter.

*NOTE*: Before adjusting the beam diameter, record or save the default beam expander settings. Ensure that the excitation wavelength is set to the desired value before proceeding with the steps below.

8 Adjust the beam diameter from wide to narrow while monitoring the beam position on the target. The beam diameter should change, but the peak intensity should remain centered on the target (the beam position should stay within a ±0.5 mm range from the center of the target).
9 Narrow the beam diameter as much as possible.
10 Stop live mode and/or close the excitation path shutter.
11 Install the microscope objective and position the thermopile power meter ∼1 mm below the objective lens.
12 Open the shutter and/or start live mode using the scanner and power settings defined in part 1.
13 Monitor the laser power under the objective using the power meter.
14 Adjust the beam diameter until the measured power under the objective decreases by 10%‐15%. The default beam expander setting should lie within this range of beam diameter adjustments. If it does not, the beam diameter is not yet optimized for the chosen wavelength (830 nm) and objective combination, resulting in suboptimal imaging resolution. In that case, use the new beam diameter setting for both track ablation and imaging settings at 830 nm. Keep the power meter and the objective mounted on the microscope and proceed to part 3.

#### Part 3: Laser power calibration

Accurate measurement of the laser power under the objective is crucial for reproducible and effective collagen track ablation. Ensuring the correct power level helps achieve consistent ablation without damaging the sample or compromising image quality.

15 Calibrate the excitation power under the objective in increments of 5% across the full laser power range. Record the laser power measured under the objective as a function of the laser power setting to create a reliable calibration curve for precise power control during experiments.
16 Stop the live mode and/or close the excitation path shutter, and then carefully remove the power meter.
  As an outcome, this calibration provides an accurate reference to set and reproduce the desired laser power under the objective, ensuring consistent ablation efficiency and imaging quality throughout experiments.

## Support Protocol 3 GENERATION OF STACKS OF LONG TRACKS BY THREE COMMON MICROSCOPE TYPES

Here, we describe scanner settings and the generation of multiple tracks for the three most commonly used microscopy systems, from Zeiss, Miltenyi Biotec, and Leica (supporting Basic Protocol 3, steps 13 and 15). Finally, we provide details on the hardware and settings of our TriMScope‐II multiphoton microscope (Miltenyi Biotec), which was used for the measurements presented in this protocol.

### Track generation using Zeiss (Zen Blue) or Miltenyi Biotec (ImSpectorPro) software

The tiling mode of the microscope software can be used to stack tracks. However, tiles in a tile region overlap in both directions. Therefore, the following workaround must be applied to ensure that tracks remain separated from each other (Fig. 6B, lower row):

1a. Follow the procedure for generating long tracks in the main protocol for track ablation (Basic Protocol 3, step 15).
2a. Increase the ablation region width to match the desired center‐to‐center (heart‐to‐heart) spacing between tracks. For example, setting the width to 30 µm will result in parallel tracks spaced 30 µm apart along the *y* axis.
3a. Define the number of stacked tracks by adjusting the *y* parameter in the tiling interface. For instance, set *y* = 10 and *x* = 4 to create 10 vertically stacked tracks, each composed of 4 consecutive, overlapping ablation regions (Fig. 6B).
4a. Convert the tile region into a list of individual tile positions:
*Zeiss Zen Blue interface*: Right‐click on the tile region, select “convert to positions,” “as single positions.”
*Miltenyi ImSpector interface*: In the *xy* stage window, on the mosaic tab, click the button with the matrix of squares. The mosaic will be converted to individual positions on the position tab.
5a. In the scanner settings, reduce the ablation region width to match the intended track width, and ensure that this update is reflected in the software's tiling interface. This adjustment will separate tracks in the vertical (*y*) direction while maintaining overlap between adjacent horizontal positions (*x*). This causes continuous horizontal tracks to be preserved, provided that stage and sample drift remain minimal.
6a. Optimize the scan order of the tile list so that positions corresponding to the same track are processed sequentially (i.e., by row; Fig. 6B).
7a. Initiate the automated routine to perform ablation across all tile positions.

#### Track generation using Leica software (LASX with Navigator)

Use the following procedure to stack tracks:

1b. Load the “inspection” image settings.
2b. Open the LASX Navigator module. Use the “spiral scan” mode to acquire an overview image of the area where the tracks will be positioned.
3b. Load the “ablation” microscope settings.
4b. In the “Stage” tab of the “Navigator” module (on the left), set the tile overlap to 15%.
5b. Draw an *x*,*y* tile region, with *x* = 1 and *y* as the desired integer length of the track (i.e., the number of vertically stacked, overlapping ablation regions). This tile region should be oriented vertically in the Navigator interface.
  The orientation of the images in the Navigator matches the physical orientation of the sample on the stage. However, the image shown in the Acquisition tab is rotated 90° to align the fast scanning axis horizontally.
6b. Reposition the tile region so that the first (or last) tile extends halfway outside the collagen border, while the remaining tiles stay fully within the collagen area.
7b. Save the tile region and reopen the generated file using a text editor (e.g., Notepad). Copy and paste the track location multiple times (e.g., 10 × for 10 stacked tracks), and modify the *x* coordinate of each duplicated entry to define the spacing between adjacent tracks.
  Add an incremental shift (e.g., multiples of 30 µm for a heart‐to‐heart track distance of 30 µm) to the x coordinate of each successive region.
8b. Load the modified tile region file into the software.
9b. Start the automated procedure to perform ablation at all tile positions.
10b. Reload the “inspection” image settings and verify that the tracks have been successfully ablated.

#### TriMScope‐II hardware and settings used for the measurements shown in this protocol paper

Track ablation and imaging as presented in this paper were performed with a multiphoton microscope (TriMScope II, Miltenyi Biotec, Germany) equipped with a tunable femtosecond Ti‐Sapphire laser (Chameleon Ultra II, Coherent Inc. USA), a high‐NA, long‐working‐distance water objective (CFI75 Apochromat 25XW MP1300, MRD77225, Nikon, Japan), and a motorized stage (Intravital stage, Miltenyi Biotec). The microscope was operated with software and a Windows computer supplied by the microscope vendor (ImSpectorPro, Miltenyi Biotec). Ablation was performed with a scanning frequency of 50 Hz, a pixel dwell time of 21 µs, a pixel size of 0.32 µm, and a laser tuned to 830 nm with a power of ∼300 mW under the objective. SHG emission was generated with similar settings, except for scan frequency (800 Hz) and power under the objective (35 mW). Emission was collected in forward direction with a 1.4‐NA condenser (Evident Inc., Japan), bandpass filtered (ET417/60, Chroma Corp., USA), and detected using an alkali PMT (H7420‐01, Hamamatsu, Japan).

## REAGENTS AND SOLUTIONS

### Collagen mix

Collagen I, high concentration (8‐11 mg/ml), rat tail, 100 mg (Corning, cat. no. 354249)
10× Minimum Essential Medium Eagle (Sigma, cat. no. M0275)
1 N NaOH (VWR, cat. no. NC2024371)
Milli‐Q‐purified water
80 mM HEPES (Gibco, cat. no. 15630‐056; optional—see Table 1)
Culture medium (see recipe)
  In preparing the collagen matrix, keep all stock solutions on ice; use of low‐retention pipet tips is recommended to minimize volume loss.

### Culture medium

Dulbecco's Modified Eagle Medium with 4.5 g/liter d‐glucose, NEAA (DMEM; Gibco, cat. no. 10938‐025)
10% fetal bovine serum (FBS, also called FCS; Sigma, cat. no. F7524‐500ML)
100 U/ml penicillin/100 µg/ml streptomycin (Gibco, cat. no. 15140‐122)
2 mM l‐glutamine (Gibco, cat. no. 25030‐081)
1 mM sodium pyruvate (Gibco, cat. no. 11360‐070)

### Phosphate‐buffered saline (PBS), 1×, pH 7.2‐7.4

1 liter Milli‐Q‐purified water
8 g NaCl
0.2 g KCl
1.38 g Na_2_HPO_4_·H_2_O
0.26 g KH_2_PO_4_
  Recipe from the Radboud UMC internal Department of Laboratory Support & Media Preparation.

## COMMENTARY

### Background

All guidance models described in this article require 3D collagen hydrogels polymerized into porous networks as a basis. Typically, for in vitro research applications, type I collagen is extracted from animal tissues using a combination of purification, salt fractionation, and sterilization steps, followed by acid treatment to dissociate the collagen into monomeric subunits (Gross & Kirk, 1958). The most commonly used sources of collagen are rodent tails and bovine skin. Due to its higher degree of natural crosslinking, bovine skin collagen generally requires additional enzymatic processing, most notably pepsin digestion, to remove telopeptides and facilitate solubilization. Upon exposure to a warm, neutral‐pH environment (e.g., through the addition of bicarbonate ions or NaOH), collagen monomers spontaneously reassemble into fibrillar structures within minutes (Mosier et al., 2023; Wolf et al., 2013). Collagen hydrogels can be additionally crosslinked, for example by ribose, which impacts gel stiffness (Mason et al., 2013). The kinetics of this polymerization process depends on the collagen source, its pretreatment, and the polymerization conditions, potentially including additional crosslinking, which strongly influence the final scaffold architecture and porosity. As outcome, collagen hydrogels of ∼20‐ to 1000‐Pa stiffness are produced that are to various extents deformable by cell‐mediated traction forces (Beunk et al., 2022; Haeger et al., 2014; Wolf et al., 2013; Zanotelli & Reinhart‐King, 2018), similar to what has been observed in cancers in vivo.

Multiphoton microscopy (MPM) utilizes pulsed infrared lasers to achieve nonlinear excitation confined to the focal volume, enabling the delivery of highly localized energy at micrometer resolution while minimizing photodamage to surrounding tissue. This principle makes MPM uniquely suited for manipulating and imaging biological specimens in three dimensions. MPM supports a range of laser‐induced processes driven by high peak intensities at the focal point. These include multiphoton‐induced photobleaching and photodegradation for targeted chemical manipulation, laser ablation for microsurgery or track formation, and polymerization for hydrogel structuring and optogenetic scaffold design (Brigo et al., 2017; Konig, 2000; Oron et al., 2012). MPM also enables the generation of label‐free signals, such as second‐ (SHG) and third‐harmonic generation (THG), which can be used to visualize collagen, lipid interfaces, or other structural elements without dyes (Friedl et al., 2007; Weigelin et al., 2012). This makes the technique particularly powerful in live high‐resolution imaging and precise micro‐engineering of complex 3D environments, such as the microtracks described here. The microtrack assay can be used for tumoroid culture (Ilina et al., 2011) as well as for the study of single‐cell migration (Video 1; Beunk et al., 2022).

### Critical Parameters and Troubleshooting

This section highlights the most common and critical experimental pitfalls that can affect the performance and reproducibility of collagen‐based guidance models. We also provide practical solutions to support effective track formation and imaging. For a comprehensive overview of key parameters, possible error sources, and recommended troubleshooting strategies, see Table 2.

#### Collagen hydrogels are polymerized incompletely

Achieving homogeneous polymerization in three‐dimensional (3D) collagen gels within a certain pore size range is critical for in vitro models, particularly when quantifying parameters that are sensitive to pore size, such as cell migration rates, nuclear deformation, or cell jamming (Haeger et al., 2014; Wolf et al., 2013). Polymerization may be delayed or incomplete, or fail entirely, under certain conditions, such as reduced or absent telopeptides, suboptimal pH (<6.5), low temperatures (e.g., ≤4°C), or excessive salt concentrations (reviewed in Wolf et al., 2009). Notably, delayed polymerization is sometimes desirable, as it can lead to the formation of looser networks with thicker collagen fibrils, producing larger pore sizes that more closely mimic in vivo ECM architecture.

#### Collagen hydrogel assembly is inhomogeneous

Larger inhomogeneities in fiber density and thickness can arise when the collagen solution is not properly mixed, for instance, due to localized variations in pH. Rat tail collagen begins to polymerize rapidly, typically within 2‐5 min, which means that slow pipetting, especially by inexperienced users, can result in premature polymerization within the pipet tip, leading to non‐uniform gel formation. Additionally, overly vigorous or rapid mixing can introduce air bubbles, further contributing to inconsistencies in hydrogel density. Adhering to the handling guidelines outlined in Table 2 will support the formation of more homogeneous collagen hydrogels, thereby improving experimental reproducibility and data quality.

#### Tissue tracks are less defined or incomplete

Successful track generation depends critically on (1) a tightly focused laser beam with a small focal volume and (2) stable, sufficiently high laser power under the objective. The size and shape of the focal volume are determined by the point‐spread function (PSF), which is optimal when the laser beam is properly aligned and its diameter adjusted to match the objective back aperture (Fig. 5B; Tsai & Kleinfeld, 2009).

Misalignment of the excitation path produces an irregular focal volume, which in turn leads to poorly defined ablation tracks during the burning process. Such misalignment may originate from the laser itself, as the position, size, or angle of the emitted beam can vary with environmental conditions (e.g., temperature or humidity) or with the selection of a different output wavelength. Instability in the microscope environment, such as temperature fluctuations or mechanical vibrations, can also disturb the excitation path. It is therefore essential to maintain stable conditions in the microscopy lab. Practical measures include relocating heat‐generating equipment (e.g., laser chillers or large power supplies) to a separate room and mounting optical components on an anti‐vibration table. In addition, air conditioning with continuous (modulated) regulation, and avoidance of direct cold air drafts onto the microscope, greatly enhance laser beam stability. During alignment optimization, the laser beam should be centered and positioned orthogonally at the back focal plane of the objective (Fig. 5B). The alignment procedure depends on the microscope system: turnkey multiphoton microscopes (such as the Leica DIVE) provide automated alignment via control software, whereas older or custom‐built systems require manual mirror adjustments in the excitation path, often using irises or quadrant detectors as guides (Support Protocol 2, part 1).

The optimal PSF is achieved when the laser beam size is matched to the objective's back aperture diameter. As a compromise between maximizing spatial resolution and maintaining sufficient power transmission through the objective, the back aperture should be overfilled with a factor 1/*e* ^2^ (Tsai & Kleinfeld, 2009; Fig. 5B and Support Protocol 2, part 2). Because back aperture diameters vary across objective manufacturers and magnifications, and laser beam diameters are often wavelength dependent, beam expansion settings must be adjusted whenever the objective or excitation wavelength is changed. Correct beam alignment and effective overfilling can be verified by measuring the PSF with fluorescent microspheres, as described in the main protocol. Further guidelines on the measurement and analysis of the PSF are available from the QUAREP‐LiMi initiative (Faklaris et al., 2022; Nelson et al., 2021).

The maximum laser power (peak power per pulse) available under the objective depends on several factors, including excitation wavelength, objective properties, and pulse duration. In general, objectives with lower numerical aperture (NA) generate larger focal volumes and therefore require higher laser power to produce well‐defined ablation tracks. Excessive power, however, risks damaging optical components or overexposing the collagen sample, leading to bubble formation. To optimize track generation, objectives with NA >0.9 are recommended, together with system‐integrated features such as dispersion compensation to preserve pulse quality and reduce power demands. Substrate properties, including collagen density and fibril thickness, which vary with polymerization temperature and collagen source, also influence the required laser power. High ablation powers may destabilize the laser due to back‐reflections from optical elements in the excitation path; this can be mitigated by slight realignment of the excitation path or by adding a Faraday isolator to block reflections into the laser cavity. Detailed procedures for measuring ablation power and stability under the objective are available from the QUAREP‐LiMi initiative (Gaudreault et al., 2025), and a brief protocol for measuring excitation power is provided in Support Protocol 2, part 3. If ablation tracks appear poorly defined, systematically re‐examining key parameters—objective NA, collagen density, pulse quality, and laser stability—can help identify and correct the problem.

#### Tissue tracks are not continuous

Accurate assessment of cell migration within long, narrow in vivo‐like tracks requires perfect overlap of successive ablation tiles. Misalignment may arise from sample creep, insufficient fixation of the sample to the microscope stage, or stage inaccuracies, ultimately leading to conditions under which further cell migration is not possible, as shown in the 5‐µm‐ and 2‐µm‐wide tracks in Figure 6C. To address this issue, the ablation strategy should be optimized to minimize the effects of mechanical instability, sample drift, and stage hysteresis on tile overlap. Practical solutions include securing the sample more firmly using stage clamps or improving the fixation of the stage itself. It is also important to assess whether the sample is mounted under tension or whether systematic positioning errors, such as those caused by mechanical hysteresis in a worn stage, are contributing to misalignment. Further recommendations on mitigating sample creep and validating mechanical stability are available from the QUAREP‐LiMi initiative (Carvalho et al., 2023).

#### Tissue tracks cannot be visualized clearly by SHG

For a rapid and effective quality control of the collagen ablation process during track generation, SHG imaging provides a fast, label‐free, and high‐resolution detection method (see Fig. 6C; Friedl et al., 2007). SHG detection works best in forward direction, and backward SHG typically yields significantly weaker signals (Weigelin et al., 2012). If, however, forward‐directed SHG does not produce a strong signal with a high signal‐to‐noise ratio, verify the presence and alignment of a high‐numerical‐aperture (NA) condenser in the transillumination path. The condenser should be positioned close to the sample, centered in the optical field, and the aperture diaphragm fully opened to maximize SHG signal detection. If forward detection remains sub‐optimal, SHG can alternatively be collected in the epi (backward) direction. If backward detection is required, image quality can be improved by placing a reflective surface (e.g., aluminum foil) beneath the sample to enhance SHG backscattering. Alternatively, ultrasensitive detectors, such as GaAsP photomultiplier tubes, hybrid detectors, or solid‐state silicon detectors, can be used, provided that appropriate precautions are taken to prevent damage during the ablation process.

### Understanding Results

**Figure 7:**
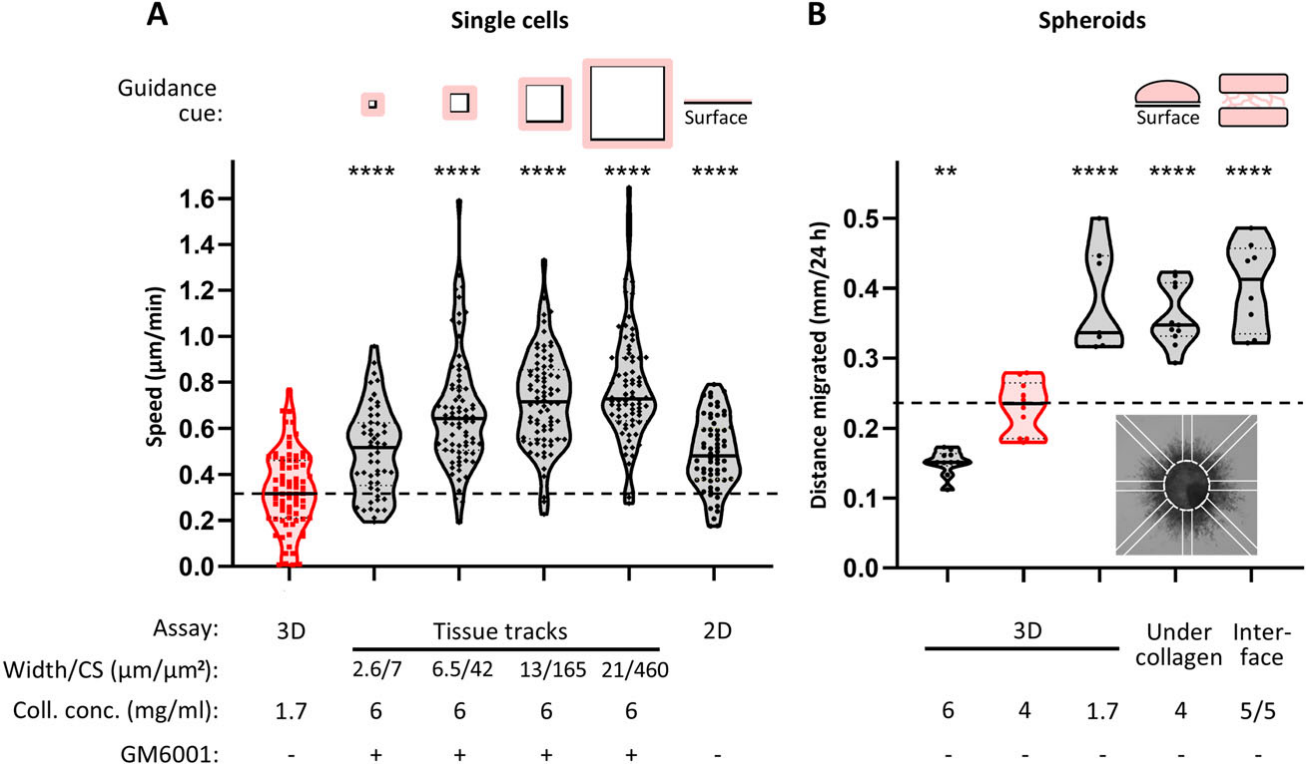
Migration behavior of HT1080 fibrosarcoma cells across different collagen‐based models for invasion guidance. HT1080 cells were detached and added to the collagen mix, here of rat tail origin, either as suspended single cells (**A**) or as pre‐formed spheroids (**B**). Where indicated, the MMP inhibitor GM6001 (5 µM) was added to the assay. (**A**) Averaged single cell migration speed, with each dot in the violin plots depicting a cell. Cell migration was monitored during live‐cell microscopy over 24 hr in different migration assays with different collagen concentrations as specified. For all single‐cell assays, *n* = 3. In the 2D assay (at right), not described in this article, cells in FBS‐containing medium were attached on top of a glass coverslip and their migration speed was measured. CS, cross‐section. (**B**) Spheroids in collagen were incubated over 24 hr, fixed in paraformaldehyde, and imaged and quantified as shown in the inset. The distance of the migrated cells from the spheroid core (dashed ellipsoid) was determined by measuring the average migration distance from 8 positions radially from the core in the different indicated models. Each dot depicts a single spheroid. For all spheroid assays, *n* = 3, with 8‐11 spheroids measured per condition. Horizonal solid lines indicate medians; horizontal dotted lines depict the 25th and 75th percentiles. The values from the randomly spaced 3D collagen assay, depicted in red, form a reference for the guided assays. **p < 0.01; ****p < 0.0001. One‐way ANOVA followed by Dunnett's multiple‐comparisons test.

Guidance models aim to recapitulate the anisotropic features of heterogeneous ECM environments in vivo. In our work, we quantitatively assessed how guidance cues of different, in‐vivo‐like geometries influence HT1080 cell migration efficiency (Fig. 7, Table 3). Thus, we tested the same cell type across various collagen‐based assay systems. As a reference, the migration of single cells and collective emigration from spheroids into a randomly polymerized 3D collagen hydrogel lacking directional cues was analyzed (Fig. 7; red violin plots). These isotropic conditions served as a baseline for comparison in determining how structural anisotropy modulates migration efficacy.

**Table 3:** Impact of Collagen‐Based Guidance Models on Cell Speed

ECM geometry/model	Structural characteristics	Locomotion efficacy above 3D random collagen	Key observations/interpretations
Random 3D collagen	Isotropic fibril network; no directional cues	Baseline	Serves as baseline for assessing anisotropy effects in the collagen model.
Flat 2D surface	Unconfined, planar substrate	↑ ∼50%	Absence of structural barriers enhances migration; speed depends on substrate stiffness and ligand density.
Linear track, 2.6 µm width	Narrow channel; strong confinement; strong cell deformation (∼80%)	↑ ∼65%	Linear guidance enhances migration; but when compared to wider tracks, migration efficiency is impeded by strong confinement.
Linear track, 6.5 µm width	Channel with some confinement; moderate cell deformation (∼50%)	↑ ∼105%	Linear guidance enhances migration; only mild slowdown occurs under moderate confinement.
Linear track, 13 µm width	Tube‐like collagen channel matching cell size	↑ ∼125%	Track cross‐sections are optimal relative to cell size. Focal adhesions and actin filaments align with track walls, promoting fast migration.
Linear track, 21 µm width	Wide track exceeding cell diameter	↑ ∼130%	Cells remodel soft walls to match body width, maintaining efficiency.
Under collagen assay	Flat surface beneath dense 3D collagen “lid”	↑ ∼50%	Cells navigate between, degrade, or lift fibrils connected to the surface.
Interface cleft assay	Boundary between two dense collagen lattices	↑ ∼75%	Cleft enables rapid spheroid emigration despite collagen bridges; migration is not dependent on stiff substrate.

The data and interpretations presented here summarize the key findings referring to Figure 7 and the Understanding Results section.

#### Impact of flat or cleft‐like ECM guidance features on cell migration efficacy

When migrating on a flat surface or within a cleft, mesenchymal cells, such as fibroblasts or the HT1080 fibrosarcoma cells tested here, spread and flatten to a height of just a few micrometers (typically 4‐5 µm). Under vertical confinement, mesenchymal cells can further reduce their height, provided that there is sufficient lateral space for extension. In line with the concept that guidance cues promote migration, we observed that 2D migration on the isotropic surface of a cell culture dish, absent any physical barriers, was enhanced by ∼50% compared to single‐cell migration in 3D randomly polymerized collagen (Fig. 7A). Although it appears intuitive that the absence of structural impediments enhances migration speed, substrate stiffness and ligand presentation greatly influence 2D migration efficacies (Doyle et al., 2009; Palecek et al., 1997; Ulrich et al., 2009). Likewise, migration along the coated cleft between the culture dish and a 3D collagen layer (4 mg/ml) supported 50% faster migration compared to migration into randomly organized 3D collagen (Fig. 7B). This suggests that the moving cells can near‐freely navigate between, or even lift, the collagen fibrils connecting to the 2D surface. Cleft formation via an interface between two dense 3D collagen lattices further enhanced spheroid emigration by up to 75%, despite low‐density collagen fibrils bridging the two compartments (Fig. 1D, arrowhead) and the absence of a stiff underlying substrate. Interestingly, Beunk et al. (2023) demonstrated that MV3 melanoma cell migration on the surface of a 3D collagen lattice increased by 300% when compared to interface migration, highlighting that in the absence of structural barriers, cell migration can be significantly enhanced.

#### Impact of tube‐like ECM guidance features on cell migration efficacy

Mesenchymal cells, such as fibroblasts or HT1080 fibrosarcoma cells, adopt a spindle‐like shape when migrating in 3D ECM, with diameters ∼10 µm (range: 8‐12 µm) and cross‐sectional areas of ∼75 µm^2^ (50‐100 µm^2^) (Wolf et al., 2013). Migration is efficient when the available substrate space matches these dimensions or when HT1080 cells are allowed to locally degrade ECM through matrix metalloproteinase (MMP) activity, providing optimally sized space (Wolf et al., 2013). Yet, when compared to migration in such 3D collagen matrix, cells migrating in 10‐ to 13‐µm‐wide (∼100‐ to 150‐µm^2^ cross‐section) linear tracks showed an ∼125% increase in speed (Fig. 7A). When track diameters were reduced to ∼6 µm or ∼3 µm (40 or 7 µm^2^ cross‐section, respectively), both cell and nucleus underwent greater deformation (∼50% or 90%), yet migration speed was reduced only moderately, by 10% or ∼30%, respectively (Fig. 7A; Beunk et al., 2022). In contrast, in 3‐µm‐size *porous* matrix in the presence of MMP inhibitors, the migration of MDA‐MB‐231 and HT1080 tumor cells was fully blocked (Wolf et al., 2013), highlighting the importance of a deformable linear track for guided migration. This directional advantage likely reflects the ability of focal adhesions and actin fibers to align along the track axis (Balzer et al., 2012; Ray, Lee, et al., 2017), forming a polarized migration unit that supports forward movement under confinement. Interestingly, 20‐µm‐wide tracks support migration just well as 10‐µm tracks, despite offering a less matching width to the polarized cell body. This can be explained by the observation that cells laterally pull on the wide track walls, remodeling the soft collagen matrix to their diameter and effectively tuning the confinement to match their needs (Beunk et al., 2022).

#### Conclusions

These side‐by‐side comparisons across the different models provide insight into how geometric features, such as barriers or directional guiding cues, affect cell migration efficacy. In the aggregate, the migration data obtained in topologically controlled environments indicate that (cancer) cell migration efficacy is strongly shaped by tissue architecture. High speed is most effectively supported by aligned, mild cleft‐ or track‐like confinement forming linear guidance between compartments, features commonly found in vivo. Therefore, to accurately assess how tissue structure modulates cell motility, physiologically relevant 3D models are essential. The methods described here provide a useful platform to investigate migration dynamics in relevant processes, such as immune cell migration, cancer or immune therapy.

### Time Considerations

All assays described here can be prepared within a timeframe of 24 to 48 hr. Spheroid formation typically requires 24 hr but may take up to 48 hr depending on the cell line and specific assay conditions (Tronolone et al., 2024; Veelken et al., 2017). The under‐collagen and interface assays can be assembled within a few hours once spheroids or single‐cell suspensions are ready. The tissue track assay requires more time: after assembly of the silicone chamber and addition of the collagen mix, the construct should be stored overnight at 4°C to allow stabilization of the collagen hydrogel. Laser ablation is then performed on the following day. After ablation, the chamber can either be returned to the refrigerator for another overnight rest or immediately seeded with a cell suspension, depending on experimental requirements. Note that laser ablation is a labor‐intensive procedure, and initiating live‐cell imaging immediately afterward may take longer than expected. In such cases, it is advisable to seed the cells the next morning. Subsequent collagen‐cell co‐culture periods during which cells migrate and interact with the ECM typically last another 24 to 48 hr, depending on the cell line and its migratory behavior. Therefore, complete assay preparation and culture, including optional live imaging, usually spans 2 to 4 days. If the experimental endpoint involves fixation, staining, and imaging (Svoren et al., 2023), an additional 1 to 2 days should be allotted for these procedures. Thoughtful planning based on these timelines will support successful and reproducible experiments.

### Author Contributions

**Stijn den Daas**: Formal analysis; methodology; writing—original draft. **Gert‐Jan Bakker**: Conceptualization; investigation; methodology; software; writing—original draft. **Diede van Ens**: Formal analysis; investigation. **Eleni‐Andria Grosu**: Methodology. **Manon Vullings**: Methodology; supervision. **Lianne Beunk**: Data curation; investigation; methodology; supervision. **Peter Friedl**: Conceptualization; funding acquisition; methodology; project administration; resources; writing—review and editing. **Katarina Wolf**: Conceptualization; data curation; funding acquisition; project administration; resources; supervision; writing—original draft; writing—review and editing.

### Conflict of Interest

The authors declare no conflict of interest. The funding sources had no involvement in the collection, analysis and interpretation of data.

## Supporting information

Supporting Information File 1: .STEP file for fabrication of the silicone chamber used in the 3D tissue track assay.

Supporting Information File 2: .STL file for fabrication of the silicone chamber used in the 3D tissue track assay.

## Data Availability

The data, tools, and materials (or their sources) that support the protocols are available from the corresponding author upon reasonable request.
